# Preparing a technologically savvy public health workforce: an academic-industry partnership

**DOI:** 10.3389/fpubh.2026.1869077

**Published:** 2026-07-02

**Authors:** Michelle Y Ke, Kai Zheng, Tim Bruckner, Margaret Schneider

**Affiliations:** 1 Institute for Clinical & Translational Science, University of California, Irvine, Irvine, CA, United States; 2 Donald Bren School of Information and Computer Sciences, University of California, Irvine, Irvine, CA, United States; 3 Joe C. Wen School of Population & Public Health, University of California, Irvine, Irvine, CA, United States

**Keywords:** digital public health, health informatics competencies, informatics workforce, program evaluation, public health education, public health workforce development, workforce development and training

## Abstract

**Background:**

To realize the potential of using information technology (IT) and data to inform public health, it is critical that the US dedicates resources to preparing a robust workforce skilled in leveraging IT, informatics, and data science towards the advancement of public health. In the wake of the COVID-19 pandemic, several innovative educational partnerships have emerged to fulfill this mission. In this paper, we present indicators of the successful implementation of an academic-industry partnership.

**Methods:**

Students enrolled in the Public Health Informatics & Technology (PHIT) program completed surveys and interviews during and immediately following participation in a paid Health Informatics internship embedded within the program. Data were summarized annually and used to refine implementation of the PHIT internship.

**Results:**

PHIT internships were hosted primarily by non-profit (53%) and health IT (22%) organizations. Over 4 years, 149 students completed the summer internship program (79% female, 21% Hispanic, 57% first-generation college students). Satisfaction with the program was high, and students’ satisfaction with positive aspects of the internship remained stable over time and concerns about negative aspects of the internship decreased over time (*p* < 0.05).

**Discussion:**

The PHIT program successfully integrated training efforts across two academic disciplines (Informatics and Public Health) and industry partnerships. The program has yielded several cohorts of trained professionals ready to enter the public health workforce proficient at applying analytic methods to large health datasets. Improvements over time in students’ evaluations of the internship demonstrated the value of ongoing program evaluation with timely feedback provided to program managers.

## Background

A skilled workforce adept in using information technology (IT) to analyze large data sets is essential for the future of public health (1–3). The experience of the COVID-19 pandemic has demonstrated the urgent need for a new generation of public health professionals with a high level of competency in informatics (Leider et al., 2023). These workers will be crucial in modernizing the nation’s public health information systems and leveraging data to track and manage future infectious disease outbreaks (4–6). Similarly, the need to equip the 21st-century public health workforce with the skills to use digital tools such as social media and mobile health apps to engage vulnerable populations is evident. These tools can help improve public health communication, drive behavior change, and enable collection and analysis of data on social determinants of health (7, 8). Furthermore, it is crucial to build a future public health workforce that is representative of the communities they serve and includes individuals with first-hand experience as members of these communities (9). Scholars have argued that such lived experience is essential to inform all aspects of data analysis and interpretation (10). To achieve these goals, the University of California, Irvine (UCI) implemented the Public Health Informatics and Technology (PHIT) Workforce Development Program in 2021. This initiative sought to enhance as well as expand the pipeline for public health professionals to build a technologically savvy public health workforce. This program was funded by the Office of the National Coordinator for Health Information Technology (ONC).

A major component of the UCI PHIT Program is its Summer Internship Program. Health Informatics internship programs can provide students with opportunities to acquire practical experience and learn directly from industry professionals. As the use of digital public health technologies continues to grow, academic-industry collaboration in providing training through certificates and internships can equip students to meet the demands of a technology-driven healthcare environment (11). Students who have participated in Health Informatics internships have reported that the experience positively influenced their learning, interpersonal and personal skills, and employment prospects (12). However, numerous challenges can limit their effectiveness. These include unclear or undefined objectives, insufficient guidance or mentorship, poorly structured tasks, rigid deadlines, limited opportunities for skill development or advancement, a lack of networking prospects, exclusion from professional meetings, and inadequate training (13, 14). Furthermore, there are very few studies that have investigated student satisfaction with internships in health informatics (15), despite the growing need to evaluate the Health Informatics discipline regarding internship effectiveness and interns’ satisfaction (12). This paper contributes to this limited literature by not only assessing student satisfaction but also examining how systematic evaluation can be leveraged to improve program design over time. The objective of this paper was to describe the UCI PHIT program and present evidence for its successful implementation of its internship program.

## Methods

### Program description (curriculum, program administration, evaluation)

#### Curriculum

The PHIT program included two components: an undergraduate health informatics minor and a graduate certificate in public health informatics. Students in either of these programs were eligible to apply for a paid internship to develop practical experience in applying informatics to advance public health goals. The majority of these internships took place during the summer, but students were also offered an option to intern over regular, academic quarters during the academic school year. However, internship cycles that took place during the academic year inconsistently occurred and hosted a much smaller number of students (*n* = 9) when compared to summer cycles. As a result, this paper focuses on students from summer cycles, which were consistent in both their duration and cohort sizes. Supplementary Table A outlines the overall curriculum design. Undergraduate students in the program were required to complete three foundation courses and four elective courses to complete the Health Informatics Minor. Graduate students were required to complete one foundation course, one informatics technology course, and one sciences course to complete the Graduate Certificate. Between June 2022 and September of 2025 four summer cohorts, comprised of a total of 149 students, completed internships across 34 organizations. Students worked at a variety of internship settings across different types of organizations (internship type (number of students hosted)): hospitals and clinics (6), healthcare management setting (4), health IT organization (33), nonprofit organization (79), public health department/agency (state or local) (4), and other (23).

#### Program administration

The PHIT program was led by a team of faculty and staff from UCI’s Joe C. Wen School of Population & Public Health and Donald Bren School of Information and Computer Sciences. The administrative staff provided support with student recruiting, course planning, internship placement, and professional development. Program evaluation was led by the Evaluation Unit of the UC Irvine Institute for Clinical and Translational Science.

#### Evaluation design

The PHIT program evaluation utilized a mixed-methods approach combining qualitative interviews and quantitative surveys to evaluate student internships. While mixed-methods assessment and the use of qualitative research findings to inform the creation of quantitative instruments are well-established concepts, only a limited number of studies have outlined the specific process of developing quantitative tools from qualitative data (16, 17). For example, (18) described the process of converting qualitative interview data into quantitative values as a new methodological approach in research design. Additionally, there is evidence that using qualitative survey data to create a quantitative instrument is a valuable approach to developing forced-choice items relevant to a specific program and population (19).

In this study, we conducted formative interviews with 22 PHIT students in the middle of the first internship cohort to generate qualitative information about common barriers to and facilitators of satisfaction with the internships. We then used the results to inform the development of a survey instrument administered to students at the end of each internship period. A review of existing internship evaluation tools yielded some items tapping into the barriers and facilitators that were identified through the qualitative analysis (see Supplementary Table B), and these items were supplemented with original items developed for this study. Data from the surveys were summarized and reported to PHIT leadership annually to inform continuous program improvements.

According to the criteria of the UCI Institutional Review Board, this study was determined to be exempt from Institutional Review Board review.

### Data collection procedures

#### Formative interviews

Individual semi-structured interviews with 22 out of 27 students from the Summer 2022 internship cohort were conducted at the one-month point of the 10-week internship. Each student participated in a 20-min individual interview via Zoom with the UCI PHIT Evaluation Specialist, who was trained in qualitative research. The interviews were recorded and transcribed.

During the interviews, students were asked 14 core questions regarding their overall internship experience, working relationship with their supervisors, and challenges and improvement suggestions. The semi-structured interview also included nine optional questions that, when time allowed, assessed students’ perceptions of their career preparation, the Health Informatics minor, application concerns, specific skills learned, and amount of time spent working at the internship. All 22 students answered all core questions, and 10 out of 22 students answered the optional questions. The moderator guide for the formative interview is provided in the Supplementary materials.

#### End-of-internship survey

An online survey was sent via Research Electronic Data Capture (REDCap), which is a secure web application used in academic research for building and managing online surveys and databases (20, 21), to each student at the end of their internship. Email invitations to complete the survey provided students with assurances of the confidentiality of the data, and this message was reiterated at the beginning of the survey. Up to five reminder emails were sent every 3 days after the initial survey invite email to encourage participation.

### Development of the internship evaluation survey

The survey instrument used in the end-of-internship survey was developed based on the results of a thematic analysis of the qualitative data from the formative interview. The analysis process involved the following stages:

1. *Initial coding*: The interview transcripts were first reviewed in their entirety to identify key words and phrases that captured the essence of student experiences. This process yielded a list of codes attached to specific quotes. The Evaluation Specialist coded all 22 interviews using Excel, and themes were reviewed by the senior author. No artificial intelligence was used.
2. *Identification of themes*: The identified codes were grouped into broader themes that align with central aspects of the internship program. Seven key themes emerged from the data: Skill Sets and Learning, Pace of Progress and Onboarding, Supervisors and Meetings, Projects and Collaboration, Health Informatics Minor, Career Goals, and Positive Aspects.

Table 1 shows the themes, sample quotes from the interviews, and items included in the survey instrument reflective of these themes.

**Table 1:** Illustration of qualitative coding process.

Themes	Sample quotes	Example survey item
Pace	“…But it does not feel like really like overwhelming. I would say a lot of the things are pretty straightforward so far, and nothing is super crazy, like a lot of intensely coding or anything like that.”	I would’ve preferred to have a faster pace of learning
Supervision	“…But I think when we start doing actual projects, I think I want some more supervision, or just like towards, like, towards the beginning of it, so like so I know I’m doing what I’m supposed to be doing right.”	I would’ve liked to receive more supervision in my internship
Collaboration	“I guess working with other interns is really cool, seeing their experience and their backgrounds, and then just being able to work together as a group as a team.”	I felt that collaborating with other interns was helpful in my professional development
Learning	“I think that I’ve been learning from my internship so far in regard to what I want to learn”	The internship met my expectations
Positive Aspects	“I’ve been really enjoying the program so far”	I am happy with this internship program
Suggestions	“So I feel having those things would be useful or like just learning how to use some other software without like having to physically schedule a meeting with them, so I can just kind of work on my own time”	I would’ve liked more flexibility in my work hours
Health Informatics Minor	“I guess it prepared me a lot, for, like a public health aspect of it.”	The health Informatics courses that I have taken were helpful in preparing me for this internship
Career Goals	“It’s very applicable in the sense that like it just helps me, if like have the mindset of like every patient comes from a different background, and just like being very considerate of that “	I learned helpful technical/hard skills

Next, we developed 23 forced-choice survey questions based on these key themes. Items were scored on a five-point Likert scale (1 = strongly disagree to 5 = strongly agree). By basing the survey design on qualitative insights from the survey’s target respondents, this evaluation survey allowed for the collection of detailed feedback that could be quantitatively analyzed to comprehensively assess the internship program’s impact.

### Data analysis

To facilitate an overall evaluation of the internship experience, a factor analysis of the combined survey data collected from all four cohorts (*N* = 146) yielded two stable factors that were used to examine change over time. Several criteria were used to determine the number of factors and combination of items in each factor, including a scree plot of Eigenvalues (Cattell, 1966) and item loadings (22). The criterion cutoff was set at ±0.35 for the item loadings. Items that failed to load on a single factor were removed iteratively from the analysis. Based on this criterion, each of the retained items loaded most highly on one of two distinct factors. Cronbach’s alphas were computed for the two factors and items were removed if they weakened the reliability of the factor. Factor scores were computed as the means of factor items.

Change over time in the factor scores was examined using one-way ANOVA. *Post hoc* comparisons were conducted with Tukey HSD.

## Results

Table 2 shows the number of students who completed the internship in each cohort and the number of internship sites hosting students each summer. Across all summers, the students (*N* = 149) were a diverse sample, with 60% Asian, 13% White, and 15% “Other.” The majority of students (79%) were female and non-Hispanic, with 57% reporting being in the first generation in their family to attend college.

**Table 2:** Annual number of students and survey response rate.

Year	# of total students	Survey response rate (%)	# of total sites	Continuing sites	New sites
2022	27	96	4	N/A	4
2023	50	100	15	4	11
2024	35	94	16	8	8
2025	37	100	14	5	9

Table 3 shows which of the survey items were retained in the two factors. For items not retained after factor analysis, see Supplementary Table C. Factor 1, which contained 8 items, was labeled “Internship Satisfaction,” and reflected students’ evaluations of the overall experience including whether they learned technical and/or interpersonal skills, whether they would recommend the experience to a friend, and whether they would consider working for their host company. This factor had strong internal consistency (Cronbach’s Alpha = 0.89). Factor two, which was labeled “Internship Concerns,” consisted of four items that reflected dissatisfaction with specific aspects of the internship: supervision, communication from the supervisor, instructions received, and resources provided. In addition, Internship Concerns contained a single item assessing how helpful the site was in addressing student difficulties with the assigned tasks. The Internship Concerns factor also had strong internal consistency (Cronbach’s Alpha = 0.82).

**Table 3:** End-of survey items retained after factor analysis.

Survey items	Factor loadings	
	Factor 1	Factor 2
I am happy with the internship site that I worked for	0.81	
I am happy with this internship program	0.81	
I learned helpful interpersonal/soft skills	0.70	
I learned helpful technical/hard skills	0.73	
I would recommend this internship program to a friend	0.79	
If given the opportunity, I would seriously consider working for this company in the future	0.58	
The internship met my expectations	0.80	
The training that I have received through my internship was helpful	0.72	
I would’ve liked to receive more supervision in my internship^*^		0.83
I would’ve liked to receive more communication from my supervisor^*^		0.80
I would’ve liked to receive more detailed instructions in completing my assigned tasks^*^		0.75
I would’ve liked my internship site to provide more resources for my learning process^*^		0.68
I found my internship site to be helpful in addressing any difficulties that I had with my assigned tasks		0.59

*Items that were reverse-scored for analysis.

Table 4 shows the means and standard deviations for Internship Satisfaction and Internship Concerns for each of the four cohorts. Internship Satisfaction was high among students across all four cohorts, with mean scores remaining close to 4 on the 1–5 scale, where a 4 indicated a rating of “agree.” The ANOVA for Internship Satisfaction was not significant, indicating that students’ evaluations of the positive aspects of the internship program did not differ meaningfully across the four cohorts. Across all four cohorts, the means for Internship Concerns indicated that students in the earlier cohorts had some salient concerns about the program (note that items were reverse-scored for analysis, so a lower mean indicates greater concerns). Moreover, the ANOVA for Internship Concerns was significant (*p* < 0.05), and post-hoc Tukey’s tests indicated that there was a significant improvement in Internship Concerns between the second and third year (p < 0.05), suggesting that the PHIT program was successful in addressing student concerns over time through programmatic adjustments.

**Table 4:** Mean factor scores.

Factor		# of Items	S ‘22 Mean (SD)	S ‘23 Mean (SD)	S ‘24 Mean (SD)	S ‘25 Mean (SD)
1	Internship Satisfaction	8	3.84 (0.58)	3.58 (0.69)	3.97 (0.69)	3.83 (0.62)
2	Internship Concerns	5	2.60 (0.75)	2.97 (0.68)	3.12(0.71)	3.17 (0.72)
# of students/# of sites			26/4	50/15	33/15	37/14

Factor 1 ANOVA: *F*(3,142) = 2.57, *p* = 0.05, η^2^ = 0.05.

Factor 2 ANOVA: *F*(3,142) = 3.79, *p* < 0.05, η^2^ = 0.07.

## Discussion

The UCI PHIT program place a strong emphasis on offering its existing students, both in the undergraduate and graduate level, the opportunity to apply health informatics concepts from coursework in real-world settings. Since all of our interns are enrolled in either the Health Informatics Minor or Graduate Certificate with a structured curriculum, our students complete their coursework and experiential-learning over an extended period of time. This structure is designed to ensure that experiential learning is integrated with a structured academic curriculum to train students in progressive development of and application in health informatics competencies. Our program follows a more structured and extended timeline compared to other PHIT programs, which offer more intensive experiential-learning opportunities through expedited timelines and flexible pathways that may not require the completion of a health informatics credential program (23). In addition, the UCI PHIT program is unique in that it has a dedicated full-time staff member, the Field Coordinator, to effectively facilitate each internship cycle as the main coordinator and point of contact for all matters regarding the internship. This role was designed to contribute to the quality of internship experience for both students and partner organizations.

Given that the internship program is a key component of UCI PHIT’s workforce development efforts, evaluating student intern experiences is critical for understanding the effectiveness of the PHIT internship and identifying opportunities for continuous improvement. Furthermore, these findings contribute to the limited literature on health informatics internships and may provide insights that could be relevant to educators, program designers, and program evaluators seeking to strengthen workforce development and experiential learning in health informatics. Therefore, the purpose of this study was to analyze student evaluations of a summer Health Informatics internship. By using a mix of qualitative and quantitative methods, our results suggest that the internship had a positive impact on student reports of their training experience. Satisfaction with the internship remaining stable and high across successive cohorts, while concerns about the internship improved over time.

During the four consecutive years of evaluation conducted on student experience in the UCI PHIT internship program, we found that students reported consistently positive perceptions of the internship program. The specific items on the Internship Satisfaction scale tapped into both overall impressions of the internship experience as well as specific positive aspects, such as learning technical and interpersonal skills. Importantly, students’ levels of concern about negative aspects of the internship were alleviated in the later cohorts as compared to the earlier cohorts.

In between each cycle of internship evaluation for each summer, a detailed evaluation report was created, and information from this report was used by program staff to create program refinements for the next cycle. As a result of this evaluation process, there have been major efforts to improve the internship program across the years. These efforts included revising our student and internship site matching process, creating and sending sites a guidebook for hosting interns, and holding pre-internship information sessions and post-internship consortium meetings to discuss best practices and implementations of program refinements for the following internship cycle. The refinements in the PHIT program that occurred over time were informed by the qualitative interviews conducted with students. In the first year, students reported that they would have liked to rank their top choices of internship sites during the matching process. For the subsequent 2 years, we revised our matching process to not only implement a ranking system for students but also to give prospective internship supervisors the opportunity to meet and interview students prior to internship placements to ensure better matches between both parties.

The most substantial changes were implemented between the second and third year of the internship program. In the second year, students reported that the processes of achieving internship goals and setting project expectations could be improved. In response, an information session was organized and held by UCI PHIT staff before each internship cycle to discuss best practices in defining and achieving internship goals with students and site supervisors. These sessions were subsequently held before each cycle to ensure sites had well-structured processes of defining and achieving project objectives. This program refinement aligns with current research that highlights the importance of structured programs to prepare supervisors to support student interns effectively in health information management internships (24). In addition, students from the second cohort reported that they would have liked to learn more technical skills in health informatics during the internship. In response to this feedback, the UCI PHIT program hosted an annual meeting with internship site supervisors before each subsequent internship cycle, at which sites were invited to discuss strategies for successful internship supervision. One key focus of these discussions was centered on making sure that the majority of the assignments given to interns would advance the interns’ technical knowledge in the field. In addition, a guidebook was created to outline detailed information regarding best practices in hosting an effective internship, which aimed to enhance supervisors’ ability to support interns. This guidebook was sent to sites before each subsequent internship cycle (publicly accessible at phit.uci.edu).

While our data are consistent with improved program administration over time, several limitations to how the data are interpreted should be considered. The study relies on self-reported measures of satisfaction, which may be influenced by individual expectations or cohort-specific factors. Additionally, while the longitudinal design allows for the examination of trends over time, causal inferences cannot be made as there is no comparison group. Despite these limitations, this study contributes to the limited body of literature on health informatics internship evaluations by outlining how systematic evaluation-driven refinements correspond with improvements in student satisfaction. The findings suggest that internship programs may benefit from adopting iterative evaluation plans that not only assess outcomes, but also actively inform program development. This approach allows health informatics programs to create scalable and transferable structures that support consistent and high-quality training experiences across diverse student internship settings.

## Conclusion

Our data suggest that program refinements implemented after each evaluation cycle were effective in alleviating student concerns in the later PHIT cohorts, and that the interventions and changes made between each cycle improved student ratings for the next year. This finding suggests that our iterative evaluation approach may have positively impacted student experiences in a Health Informatics internship program.

Overall, these results highlight the value of continuous evaluation and refinements in health informatics internship program design. By incorporating student feedback into actionable and implemented program modifications before each internship cycle, previously identified barriers, such as the site placement process and technical skill development, were addressed. The observed increases in perceived goal attainment, training effectiveness, and supervision quality from overall student reporting suggest that targeted interventions as a result of structured evaluations in these areas not only improved program structure, but also enhanced the overall educational value of the internship experience for students planning to enter the health informatics workforce.

## Data Availability

The data analyzed in this study is subject to the following licenses/restrictions: This paper examined student evaluation data, which are confidential and prohibited from sharing outside of the UCI PHIT program in accordance with program policies on student evaluation data. Requests to access these datasets should be directed to Michelle Ke, yingk1@uci.edu.
