# Supplementary material for: Preparing a technologically savvy public health workforce: an academic-industry partnership
Source: Front Public Health. 2026 Jul 2;14:1869077. doi: 10.3389/fpubh.2026.1869077 (PMC13372960; doi:10.3389/fpubh.2026.1869077)
Supplement: Supplementary file 1 [file Supplementary_file_1.docx]

**Supplementary Materials**

**Table A. Curriculum Design of the UCI PHIT Programs**

| **Program** | **Curriculum** |
| --- | --- |
| Undergraduate Minor in Health Informatics | - 7 courses   - 3 foundational courses:     - IN4MATX 171 Introduction to Health Informatics     - IN4MATX 172 Project in Health Informatics     - IN4MATX 173 Consumer Health Informatics OR IN4MATX 174 Health Data Analytics   - 4 electives from 32 courses offered |
| Graduate Certificate Program in Health and Public Health Informatics | - 3 courses   - 1 foundational course:     - IN4MATX 295 Introduction to Health Informatics OR NUR SCI 251 Nursing Informatics   - 1 Informatics, Technology and Data Science course: 10 elective courses to choose from   - 1 Health Sciences course: 4 elective courses to choose from |

**Evaluation Interview Moderator Guide**

| **Interviewer Script:**  Hi (name), thank you so much for meeting with me for our first check-in interview! I am just checking in with you regarding your experiences so far in your internship, make sure that you are having a good learning experience, and to address any concerns or questions that you may have. Your feedback is very important to us, and it will help us to make sure that we are doing everything that we can do to help you succeed. So we are going to take your response in heavy consideration to make any changes necessary.  I also want to note that constructive criticism is also important for us to know, so if there is anything that isn't going well or that you don’t like about the internship, please feel free to let me know! We are trying to assess both what is and isn’t working for you.  I am going to record your responses for today. This recording is only for the purpose of me transcribing your response and to catch anything I didn’t type fast enough to write down. I’m transcribing this interview directly after it ends, so I will be deleting the recording within this hour. Of course, if you would like me to stop recording at any point, please feel free to let me know. I understand that if there are any concerns that you want to bring up that you may be uncomfortable to talk about while being recorded, I’m more than happy to stop recording so that we can talk about those concerns. Is it okay for me to record this conversation?  Are there any questions before we begin? |
| --- |
| **Core Questions**   1. How has your internship been going for the past month? 2. Who is your site supervisor? 3. How is your working relationship with your supervisor? 4. Can you tell me some of the things that are working well? 5. Can you tell me some of the things that aren’t working well? 6. Are there any changes that you would like to see in your supervision? 7. Do you have regularly scheduled meetings with your supervisor? 8. How often do you have these meetings? 9. Are these meetings one-on-one meetings or group meetings? 10. How do you feel about the amount of supervision that you are getting? 11. How, if at all, would you say that your internship is preparing you to achieve your goals? 12. How do you think your internship could be improved? 13. Did you feel comfortable and confident in handling your assigned responsibilities? 14. Anything else that you would like to tell us before we end the interview?   **Optional Questions (if there is extra time)**   1. How well do you feel that your Health Informatics minor prepared you for your internship? 2. In what ways did your education prepare you for your internship? 3. What is lacking in your education that did not prepare you for your internship? 4. What, if anything, would you like for the Health Informatics program to add to help you succeed as a student? 5. What, if any, concerns did you have about the internship when you applied for it? 6. What skills did you expect to learn from the internship? 7. Are you learning those skills from the internship? 8. How well is the internship allowing you to learn those skills? 9. How much time do you spend working at your internship? |
| **Interviewer close-out script:** Before we end this meeting, do you have any questions?  Thank you so much for taking the time to give us feedback! We’re going to use your response in heavy consideration to improve the program and address any concerns that you brought up. Thanks again! |

**Table B. Referenced Survey Instruments**

| **Organization/Source** | **Link to Public Resource** | **Number of Survey Items Referenced** |
| --- | --- | --- |
| Greater Des Moines Partnership (DSM) | https://www.dsmpartnership.com/filesimages/Working%20Here/PDF/EvaluatingtheInternship_Internships_81AD0D1DB1CE8.pdf | 4 |
| Northern Vermont University | https://www.northernvermont.edu/sites/default/files/2020-10/intern_self-evaluation_form.pdf | 4 |
| Society for Human Resource Management (SHRM) | https://www.shrm.org/topics-tools/tools/forms/student-internship-evaluation-form | 5 |
| Georgetown College | No longer accessible | 5 |
| Western Kentucky University | https://www.wku.edu/business/internships/documents/student-internship-evaluation.pdf | 8 |

**Table C. Survey Items Excluded After Factor Analysis**

| - My supervisor did a good job at setting goals and expectations - I had a good working relationship with my supervisor - I wish that I had more opportunities to collaborate with other interns - My supervisor used digital communication effectively to support my internship (e.g., email, Slack) - I found my meetings with my supervisor to be helpful - I would’ve liked for my internship site to provide more resources for my learning process - I would’ve liked more flexibility in my work hours - I would’ve preferred to have a faster pace of learning - I felt that the work that was assigned to me was health informatics related - I would’ve liked to receive more communication from UCI PHIT staff - The health informatics courses that I have taken were helpful in preparing me for this internship - I found my assigned tasks to be enriching to do - I felt comfortable asking my supervisor for help - My internship felt disorganized - It was difficult for me to reach my supervisor |
| --- |
